# A virtual reality game for older adults’ immersive learning

**DOI:** 10.1038/s41598-025-07354-2

**Published:** 2025-07-01

**Authors:** Lee Cheng, Wing Yan Jasman Pang, Anthony Kong

**Affiliations:** 1 Cambridge School of Creative Industries, Anglia Ruskin University, Cambridge, UK; 2 Department of Cultural and Creative Arts, The Education University of Hong Kong, Hong Kong, China; 3 School of Design, The Hong Kong Polytechnic University, Hong Kong, China

**Keywords:** Aging, Digital game-based learning, Cognitive skills, Life skills, Immersive technology, Serious game, Therapeutics, Health care

## Abstract

This article presents a feasibility study that assessed the effectiveness of a virtual reality (VR) game in training older adults’ cognitive and independent living skills. A home-like virtual environment, comprising six mini-games, each designed to target a specific type of learning, was created to enhance the gamified experience. A qualitative approach was used to evaluate the feasibility of the gamified approach, involving semi-structured interviews with older adults (*N* = 30) who had played the VR game. Findings revealed positive feedback on the VR game’s effectiveness in facilitating learning among older adults, including ease of use and perceived usefulness which informed their acceptance of the technology, as well as the competency and cognitive developments afforded by the VR game. The gaming experience also offers varying degrees of stimulation and engagement, although some negative experiences, such as cybersickness and anxiety, were identified that may require further attention. The study’s findings offer insights into the feasibility of employing digital game-based learning for older adults within an immersive learning environment and provide best practices for designing VR games that promote the development of cognitive and independent living skills.

## Introduction

Recent advancements have made immersive technology more affordable and accessible, unfolding possibilities for what learning is and how it can occur within virtual environments. It had led to the conceptualization of immersive learning, a concept that describes learning activities initiated by a medially enriched environment that evokes a sense of presence^1^. Catalyzed by the phantomization and democratization of software and game engines^2,3^, many innovative digital solutions have been developed in line with this concept, including the use of virtual reality (VR) games for simulated training^4^, the reconstruction of historical sites for mobile heritage experiences^5^, and the development of wearable augmented reality (AR) designs for wellbeing and healthcare^6^. These applications often incorporate elements of serious games, leveraging the stimulating effects of gameplay to increase the learners’ motivation^7,8^, thus offering opportunities for value-added informal learning experiences.

Perhaps due to the concerns related to health and safety issues^9^ and the negative stereotype that older adults are less willing to accept and less capable of using technology^10,11^, there has been less effort to advocate immersive learning among older adults^12^. However, prior studies have highlighted the potential benefits of immersive technologies in enhancing older adults’ cognitive abilities and independent living skills^13,14^, both of which are prioritized learning needs for this population^15^. Building upon the premise that immersive technology has the potential to empower older adults by mitigating age-related decline and enhancing their independence, coupled with the research team’s efforts to assert its technology acceptance by older adults^16^, this paper presents the design of a VR game tailored to the immersive learning experience of older adults. Additionally, an evaluative study is presented, assessing the feasibility of developing their independent living and cognitive skills within a home environment simulation.

## Literature review

### Older adults’ independent living and cognitive skills

Mack et al. conducted semi-structured interviews with community-dwelling older adults to gather their perspectives on crucial factors for successful independent living^17^. The study identified two major threats: the loss and maintenance of their capability to engage in specific activities and stay healthy. A survey study conducted by Parra-Rizo and Sanchis-Soler revealed the relationship between older adults’ physical activity and their ability to live independently, finding that high levels of physical activity are necessary to improve functional skills in old age and help individuals address daily living difficulties^18^. Highlighting the importance of cognitive ability, a cross-sectional study by Federman et al. demonstrated a strong association between memory and the health and independence of older adults^19^. A meta-synthesis conducted by Lommi et al. revealed the importance of self-care in older adults’ wellbeing, prevention and treatment of aging effects, life satisfaction, and self-realization^20^. These studies revealed challenges that affect older adults’ ability to live independently, including physical conditions that limit mobility, cognitive decline, and behavioral health issues such as isolation and loneliness. This combination of challenges can negatively impact their life satisfaction, functional ability, and overall social and mental health wellbeing.

### Immersive learning for older adults

The development of immersive technologies for older adults’ learning and capability building has primarily centered around addressing aforesaid aging-related concerns, such as physical health, cognitive functioning, and independent living. In a feasibility study, Appel et al. investigated the use of VR technology as a therapeutic tool for older adults with cognitive or physical impairments^21^. Participants felt more relaxed and adventurous after being exposed to 360-degree video footage featuring various nature scenes, indicating that it was both feasible and safe for them to engage with immersive environments through VR technology. Man et al. explored the effectiveness of VR-based memory training for older adults with questionable dementia in a randomized controlled trial^22^. The findings revealed that participants who received memory training tasks within virtual space showed greater improvement in objective memory performance than the control group who received therapist-led memory training, suggesting the effectiveness of VR-based intervention comparable to the conventional therapeutic methods.

Dobrowolski et al. conducted a research study to assess the efficacy of cognitive skills training for older adults in an immersive virtual environment^23^. They utilized standardized cognitive tasks and compared the transferability of the learned skills to the real world by replicating these tasks within the VR environment, revealing higher accuracy and fewer timeouts among participants in the experimental group. Building on prior studies that explored the impact of simultaneous exercise and cognitive engagement on enhancing brain function, Sakhare et al. examined the feasibility of cycling in an immersive spatial navigation VR environment for older adults^24^. Apart from the feasibility aspect, older adults reported enjoying the combination of physical and cognitive activities. Another study by dos Santos et al. focused on designing an immersive VR system for evaluating route learning among older adults^25^. The study yielded positive results regarding its applicability and short-term stability.

### Gamified immersive design for older adults

The stimulating effects of gameplay, coupled with the lifelike virtual environments made possible by immersive technologies, have contributed to heightened learner engagement^26^. Rendon et al. conducted a randomized controlled trial to assess the feasibility and outcomes of improving the dynamic balance of older adults through the use of VR gaming balance board^27^. The trial resulted in significant improvements in the experimental group in terms of dynamic balance, postural stability, and confidence with functional activities, ultimately reducing the risk of falling. These findings were supported by de Vries et al.^28^, whose study identified game mechanics as a potential source of effective muscle training stimulus for older adults. Xu et al. conducted a survey study examining older adults’ gaming experiences with commercially available VR exergames^29^. Apart from the confirmed technology acceptance, the findings also revealed the potential of VR exergames to support older adults in enhancing their physical abilities, maintaining their cognitive level, and living independently. Wais et al. developed a VR spatial wayfinding game as a cognitive intervention for older adults with declining memory functions^30^. The quasi-experimental study showed improvements in high-fidelity memory, highlighting the potential restorative effects of VR games for addressing long-term memory decline in otherwise healthy older adults. Thapa et al. conducted a randomized control trial to assess the effects of a VR-based intervention on older adults with mild cognitive impairment^31^. They designed VR gaming content to enhance participants’ attention, memory, and processing speed levels, leading to significant improvements in cognitive, physical, executive and brain function among the experimental group. Huang explored the effectiveness of VR-based cognitive training through exergames for older adults, with a pre-post-test study revealing significant effects of the VR-based intervention^32^. The sense of presence was found to be crucial for improving older adults’ cognitive performance. Barsasella et al. conducted a randomized controlled trial to investigate the influence of VR experiences on older adults’ quality of life, happiness, and functional fitness levels^33^. The intervention group exhibited significant improvements in happiness and functional fitness through questionnaire surveys and fitness tests, suggesting that VR has the potential to enhance the wellbeing and physical fitness of older adults, promoting healthy and active ageing.

## Conceptual framework

Prensky introduced the concept of digital game-based learning (DGBL)^34^, an approach that effectively blends interactive entertainment with learning, achieving a balance between the two. By merging the engaging and interactive elements of digital games with educational content, DGBL can motivate students to stay focused during the learning process. Since its inception, DGBL has served as the conceptual foundation for serious game design and research studies targeting diverse audiences, ranging from early childhood to older adults^35–38^.

The incorporation of VR into DGBL immerses learners in a virtual learning environment, impacting the learning process in different ways. The dynamics and relationships among these influencing factors are elucidated in the educational framework for immersive learning (EFiL), as proposed by Dengel and Mägdefrau^1^. EFiL encompasses a range of technological and individual factors that shape the learning experience in immersive environments, including immersion, presence, motivational factors, cognitive factors, emotional factors, and learning outcomes. EFiL serves as a theoretical framework for gaining a fundamental understanding of immersive learning, offering valuable insights into the nature of each factor and its role in the learning process. When applied in the context of DGBL, EFiL can be used to analyze various dimensions of the immersive and gamified learning experience, such as the impact of different play modes, usability in teaching and learning various subjects, and technology acceptance^39^.

Addressing older adults’ age-related physical and cognitive conditions, Guzmán et al. proposed a conceptual framework for the development of serious games aimed at cognitive rehabilitation in older adults^40^. Taking into account age-related cognitive changes, physical abilities, and sensory perception, the framework emphasizes a user-centered design to enhance the effectiveness and usability of serious games for older adults. Their framework supplemented EfiL for catering to individual needs of older adults in addition to considerations in immersive learning, providing a structured approach to designing and evaluating serious games in the current study.

## Game design

**Fig. 1:**
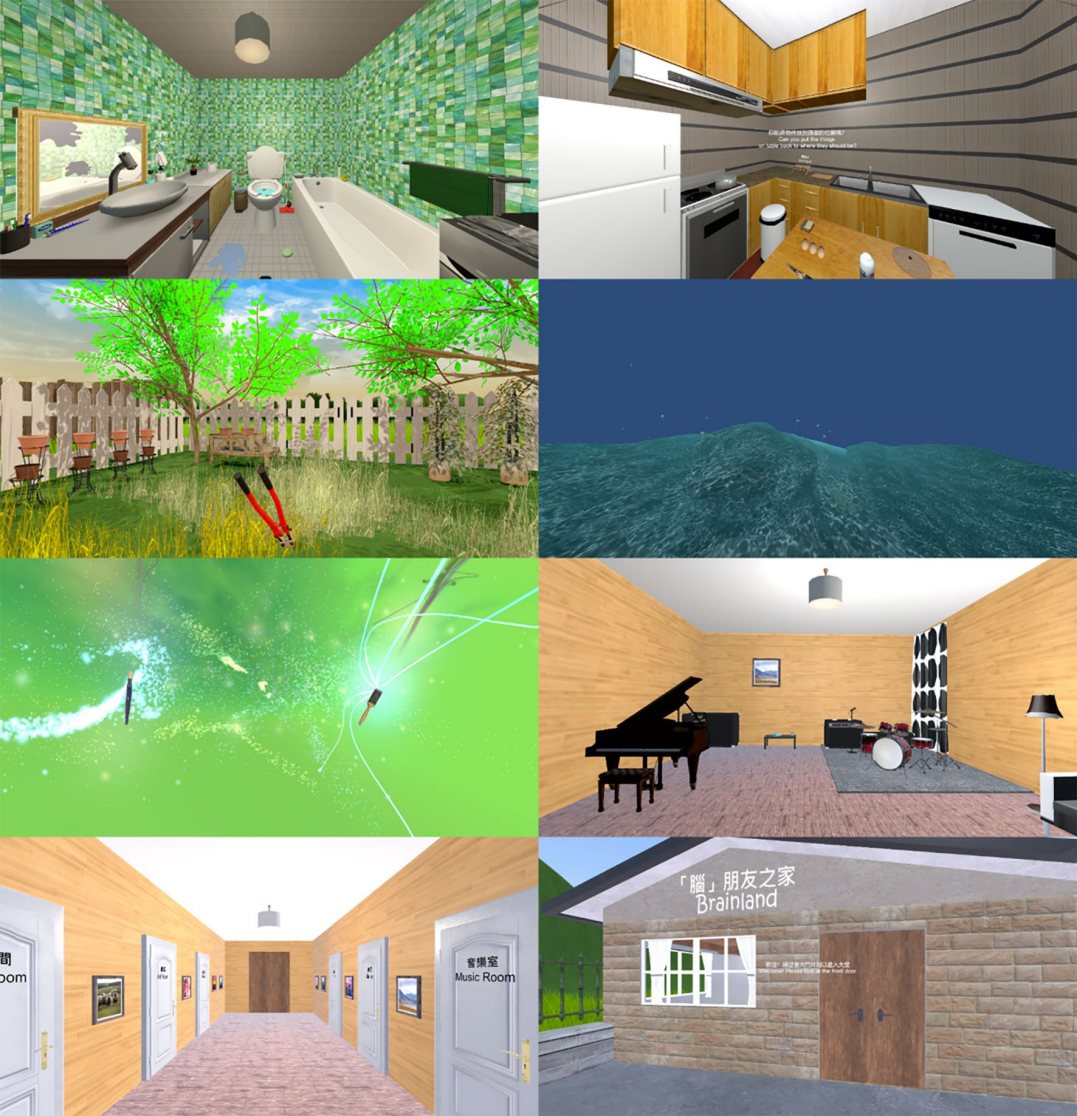
Snapshots of the six rooms in the VR game *Brainland* left to right, top to bottom: Bathroom, kitchen, backyard, swimming pool, art room, music room, hallway, entrance.

Titled *Brainland*, the present study’s VR game is specifically designed to enhance the immersive learning experiences of older adults. *Brainland* is set in a simulated home environment, a familiar and safe space for older adults, where they can comfortably engage in gameplay^41^. Players can explore various rooms and participate in home-related tasks, such as cleaning a bathroom and a kitchen, creating art in an art room, identifying the source of sounds in a music room, gardening in a backyard, and swimming in a pool (Fig. 1). These gaming tasks are designed to improve older adults’ physical health and cognitive functioning^42^, while also providing opportunities for leisure and practice self-care^15,43^. Each gaming task targets specific cognitive or independent living skills: cleaning and maintaining an organized home environment to help older adults replicate everyday activities and actively participate in society^44^; swimming as a physical activity aimed at enhancing cognitive functioning^45^; and engaging in artmaking to inspire older adults’ creativity and enhance cognitive performance^46,47^. The design also introduces features that enhance the immersive nature of the gaming experience, such as positioning the player at the center of the backyard and allowing changes in swimming direction based on hand movements.

Recognizing that some older adults may experience functional diversity in terms of finger movements, the game design incorporates the use of ‘Tracker’ from *HTC Vive* as the gaming controller. The ‘Tracker’ is a unique type of buttonless VR controller that can be attached to a player’s arm, eliminating the need to hold it. The primary focus of the game’s design is accessibility. For example, the action of opening a door in the game involves the player gazing at the targeted door for one second, with a timer displayed on the reticule. Similarly, pointing at an item signifies the player’s intention to pick it up to perform an action

## Aim and research questions

A feasibility study was conducted to assess the effectiveness of the VR game in enhancing the cognitive and independent living skills of older adults. This exploratory study aimed to evaluate the user experience (UX) and determine the perceived feasibility of capacity building through immersive gamified learning among older adults. The study was guided by the following research questions:

1. How do older adults perceive immersive learning in a gamified VR environment?
2. What cognitive and independent living skills can be acquired through gameplay?
3. To what extent are these skills relevant and transferable to the real-world environment?

## Methods

The study adopted a qualitative approach to investigate the feasibility of enhancing cognitive and independent living skills training through the VR game, as perceived by older adults (*N* = 30). A qualitative approach allows for the exploration of findings that go beyond mere description, enabling an in-depth analysis of the environment, events, behavior and other dynamics within their respective contexts^48^. This method generates rich and detailed data that can capture the complexity and diversity of people’s experiences^49^, as was the case in this study, which delved into the older adults’ learning experiences in the immersive gaming environment.

### Participants and procedure

Convenience sampling was used to select participants for this study, involving a total of thirty older adults who were approached through the personal connections of the research team. Thirteen of the participants were male and the rest were female, with ages ranging from 65 to 80 (*M* = 71.63, *SD* = 5.49). All participants were born and raised in Hong Kong. They had experiences using various technologies such as smartphones (*n* = 29), computers (*n* = 8), tablets (*n* = 9), and smartwatches (*n* = 7). None of the participants had underlying health conditions that might affect their VR gaming experience. Once participation was confirmed, suitable dates, times and venues for the trial session were arranged.

During the trial session, participants received a briefing on VR technology, including information about potential hazards and safety measures associated with the immersive experience. Ethical clearance was obtained beforehand from the Human Research Ethics Committee at the university where the second author were based. Confidentiality and anonymity were ensured, with participants provided an information sheet and consent form. They were also informed of the voluntary nature of their participation.

A team member supervised the trial session, providing instructions to the participants on how to use and adjust the VR equipment before allowing participants to explore the VR game for a duration of 20 minutes. To ensure the VR experience did not cause any discomfort or harm, participants were asked at 5-minute intervals whether they felt dizzy or experienced any discomfort. After the gameplay, participants took part in an interview session that lasted for 25 minutes. Their anonymity and the confidentiality of the information provided were assured. The interviews were audio-recorded and subsequently transcribed for further data analysis.

### Semi-structured interviews

A series of semi-structured interviews provided participants with the opportunity to offer detailed, unfiltered descriptions of their subjective understanding of the phenomenon under study^50^. A set of questions was designed to guide these semi-structured interviews. Participants were asked about their experiences during gameplay, with a particular focus on the immersiveness of the VR game and how it contributed to their learning. They were also encouraged to discuss what they had learned through completing game tasks and how these experiences may help in developing their cognitive and life skills. The interview questions are as follows:

1. How do you feel about the gaming experience?
2. How realistic do you find the virtual home environment?
3. What difficulties did you find when completing the game tasks?
4. How relevant were those tasks to your daily life?
5. What have you learned during the gameplay?
6. How relevant are the mini-game tasks to your daily life?
7. Other than the aforesaid skills, what have you learned?
8. How the immersive environment helped you to learn?
9. How relevant are these learned skills to your daily life?

### Data analysis

The data analysis, driven by the research questions and open coding of the data^51^, employed both deductive and inductive approaches. Each member of the research team carefully read through the interview scripts, labelled them with initial codes, and compiled a set of recurring themes related to the research questions through a literal interpretation of the text (Table 1). The process was repeated for each transcript until no further themes could be identified, achieving thematic saturation. Any coding discrepancies were reconciled among the team through discussion. Intercoder agreements were made among the research team in order to ensure the reliability of the findings^52^. Coding discrepancies were resolved through iterative discussion until the Krippendorff’s alpha exceeded 0.8^53^.

**Table 1:** Coding table used to analyze participants’ immersive gaming and learning experience.

Category	Code	
Technology acceptance	Easy Difficult Practice	Dizzy Comfortable Simulate
Gaming experience	Realistic Exciting Stimulate	Engage Success Challenge
Skills development	Toilet Kitchen Swimming	Gardening Cleaning Tidy
Cognitive development	Think Move Smart Brain Muscle	Hand Creative Direction Focus Logic
Environment	Similar Familiar Home	Family Daily life Real world

### Ethical approval

This study and its protocols were approved by the Human Research Ethics Committee at The Education University of Hong Kong. All methods were carried out in accordance with relevant guidelines and regulations. Informed consent was obtained from all participants via an information sheet and consent form, along with being informed about the voluntary nature of their participation.

## Results

### Immersive gaming experience

The participants found it easy to handle VR technology, adapt to the immersive environment, and familiarize themselves with the gaming controls. Some participants, particularly those with less experience using technology, initially struggled but were able to manage the technology once they became accustomed to it.

It was not difficult to use after several tries. (Participant K)It was easy to manipulate and use, even without assistance. I didn’t really have difficulty using it, and the interface was understandable. (Participant L)It’s quite easy to manipulate and easy for me to use, even without assistance. I don’t really have difficulty using it, and the interface is quite understandable (Participant H)

Along with the ease of use, participants perceived the immersive technology as useful. They expressed positive views about using immersive technology, as reflected in their trial experience with the VR game.

I think VR can help people practice or experience something they are not familiar with. (Participant J)I believe it (VR) can enhance my standard of living for the foreseeable future. At this stage, I am still capable of hiking, kayaking, diving, and even conducting site visits in the wild, but I may not be able to do these in the future. The technology can help simulate these experiences. (Participant E)At my age, health and family are the most important things. Perhaps it (VR) can prevent functional loss. (Participant G)

However, participants also reported discomfort and constraints that discouraged them from engaging with the virtual environment for extended periods. This issue was particularly serious for older participants, who experienced dizziness, discomfort, and blurred vision.

The VR headset was uncomfortable. It was difficult to steady myself, and I sometimes felt quite dizzy. (Participant DD)I felt uncomfortable wearing the headset, especially because I was not able to sense the real-world environment. I felt unsafe not being connected to the physical world. (Participant AA)Perhaps because of my presbyopia, the screen was never very clear. (Participant Z)

Participants were satisfied with the gaming experience, enjoying the gameplay, which stimulated their interest in engaging with the gaming tasks.

The game is fun to play. I haven’t been playing games for a long time, and the experience is very different from what I played many years ago. (Participant X)Yes, it’s so exciting! This is something that I haven’t gotten into before, and I would like to play it again in later. (Participant O)

A crucial aspect of their gaming experience is the feeling of achievement from completing the gaming tasks.

I have successfully completed all the tasks in the kitchen and the toilet, so they look clean now. (Participant BB)Some of the tasks are quite challenging, but I am able to complete all of them. This makes me feel so proud of my ability. (Participant CC)

The immersive environment of the VR game enhanced engagement with the gaming experience and simulated tasks.

The environment is so real. Some of the rooms do look like those in my home. (Participant U)I feel so engaged because I can move around and see different parts of the rooms as if I am there. (Participant Y)The gaming tasks are so stimulating. I need to focus on the surrounding environment to spot the dirty bits and clean them. (Participation S)

### Development of cognitive and independent living skills

Participants highlighted the kitchen and bathroom as the locations where the learned skills were most relevant and effective. They appreciated the sense of familiarity with their real-world environment and related daily life activities.

The soap should be placed in the tray, the toilet roll should be placed in the holder, and the toilet should be flushed after use... (Participant C)Tasks in the kitchen and bathroom are fun. I am familiar with these tasks, and I know what to do. (Participant AA)It reminded me to tidy up the home when it becomes messy by putting things back in their right places. (Participant H)Some of the rooms, such as the bathroom and kitchen, are very similar to the ones in my house. It makes me feel as if I’m at home. (Participant D)I would need to sweep the floor when it is wet and which tool to use for the job... The interactive elements in the game made it easier to learn. (Participant V)

Participants also improved their hand-eye coordination while using the VR headset and controllers.

When trying to swim, you need to move both arms; if you want to turn left, then move the left hand only$\ldots$ Sometimes I can get confused with the directions. (Participant N)I try to keep myself steady when working with my hands so that I can use my eyes to focus. Here I can learn how to move my head and hands at the same time to work on more complicated tasks. (Participant M)

When asked about personal development through the immersive learning experience of the VR game, the participants emphasized motor skills training through muscle movement and body coordination. Their responses affirmed that motor function development was a significant achievement resulting from playing the VR game.

They [the gaming tasks] can make me less obtuse, and train my muscles by moving the hands around (Participant O)I think they [the gaming tasks] can help improve the efficiency of my daily life through motor control, muscle and memory training that they offer. (Participant M)It can definitely train my brain and muscles $\ldots$ If the game is associated with muscle-strengthening activities, it can help players improve their bodies. (Participant T)

Participants M and T also mentioned brain functioning and memory training, which were also echoed by others.

The soap should be placed in a soap dispenser; a new toilet roll should be placed in the holder; the toilet needs to be flushed after use . . . these are daily activities that we have to cope with in everyday life” (Participant O)The game encouraged me to remember where the household items should be placed. (Participant Z)

Motor skills and memory training were guided by logical thinking and decision-making involved in each movement, contributing to participants’ cognitive development.

I think it (the VR game) can help me become ‘smarter’ by stretching my arms and moving around. (Participant F)Playing these games requires hand movement and logical thinking, which can help muscle training and cognitive thinking. (Participant W)It (the VR game) prompted me to think about what to do next and about how things work with the appropriate tool. (Participant R)Apart from muscle training, I think the games also help cognitive training by thinking logically – like putting the items in the right place or getting tasks done. (Participant J)

Artistic expressiveness was mentioned by some participants following their experiences in the art room. They noted that the generative painting exercise, as programmed in the game, empowered them with art-making abilities and fostered their creativity even if they did not possess such skills in real life.

I haven’t drawn for a while because my hands sometimes tremble. I am now able to draw differently within the art room in the game. Although the experience is different, it helps me continue to be creative. (Participant P)I never thought that I could be that creative. I could draw a lot of things around me. (Participant T)

### Transferability to daily living

Participants responded positively regarding the transferability of the skills they learned in the VR game, aided by the close degree of familiarity between the virtual and real-world environments.

I liked playing in the kitchen and bathroom. These are the places that I spend a lot of time in at home, and they looked very similar to those, too. (Participant P)I felt successful after completing the tasks in the kitchen and the bathroom; the environments were very familiar. (Participant D)Those two rooms (bathroom and kitchen) were the most familiar to me. It felt real, both in terms of the environment and the daily life challenges. (Participant I)

However, participants were uncertain about the development of cognitive skills would transfer to their daily lives.

$\ldots$ I am not sure. Swimming in the game is different from swimming in a real pool. Other than moving one’s arms, there are other skills to manage as well. (Participant Q)I think the game oversimplifies some of the real-world challenges, thereby reducing its relevance to my daily life. (Participant T)

## Discussion

### Older adults’ immersive learning experiences with the VR game

Participants responded positively to their immersive gaming experience, noting the excitement of gameplay, the realistic feeling of the virtual environment, and the perceived usefulness and ease of use of the VR game. These factors are strong predictors of older adults’ intention to use technology^54^, contributing to technology acceptance^55^. This finding specifically affirms the acceptance of immersive gaming by older adults, extending beyond the existing body of literature, which predominantly focuses on younger age groups^56,57^. It highlights the potential of VR games to combine the stimulating effects of video games with the realistic environment offered by immersive technology, resulting in value-added learning outcomes for older adults. This also helps dispel the persistent negative stereotypes about older adults’ limited ability to adapt to emerging technologies^10,11^.

While no gender differences were found, participants with more experience using technology tended to exhibit higher acceptance and were more likely to engage positively with immersive gaming. This finding echoes previous literature, which suggests that prior experience can reduce anxiety and increase confidence in adapting to new technologies and VR applications^58,59^. Age was also identified as an influencing factor in older adults’ immersive gaming experiences, with older participants reporting more cybersickness and anxiety than younger ones. While previous studies have shown that age is negatively associated with enjoyment and positively associated with experiences of cybersickness and anxiety when interacting within immersive environments^60,61^, the findings of the current study suggest that additional precautions are needed to ensure the health and safety of individuals during their immersive gameplay. While most existing research focuses on older adults’ immersive experience in care homes or community settings with the necessary safety measures^62^, the independent use of VR headsets of older adults require further considerations.

### The VR gaming experience and older adults’ skills development

Participants reported various types of skills development through gameplay, which align with the intended outcomes of the game’s learning design. These achievements encompassed a wide range of independent living and cognitive skills, echoing previous studies that demonstrated the effectiveness of gaming and immersive environments in supporting practical and higher-order learning behaviors, along with enhanced learner satisfaction and active participation^21–25,30^. In contrast to their detailed feedback on independent living skills training, participants’ responses regarding the impact of VR gaming on cognitive skill development were less explicit. This may be due to the generally lower education levels of older generations in Hong Kong^63^, which can make it challenging for them to understand and elaborate on complex concepts^64^. When asked about their perceived cognitive achievements, participants focused more on expectations or estimates of the technology’s effectiveness rather than their gaming experiences during the 20-minute trial session. The brevity of the session might be the main reason why observable benefits of cognitive training did not emerge^65^, suggesting that further research is needed to investigate the long-term benefits of immersive gaming experiences for older adults.

The immersive gaming environment, portrayed as a home, was frequently mentioned by participants in relation to its familiarity. Both the kitchen and bathroom, in particular, were positively emphasized, while other rooms were less noted. This may be explained by older adults’ familiarity with those environments, both in general^66^ and within the context of this study, considering that most living spaces in Hong Kong are too small to include backyards or leisure rooms^67^. While environmental familiarity is crucial for learning effectiveness^16^, the design of immersive learning tools for older adults should address their living spaces and sociocultural backgrounds, along with their learning needs, to ensure a more effective learning process and greater acceptance. This is particularly relevant for older adults who need more extrinsic motivation for home-based reablement, as their abilities are mediated by the familiarity of the surrounding environment^44,68^.

### Relevance and transability of learned skills

Participants affirmed the relevance and transferability of the skills they learned in the VR game to real-world situations, aligning with the intended learning outcomes of the game design and echoing previous studies^23,69^. However, participants also questioned whether the elements of the operational workflow required to complete gaming tasks would directly transfer to real-world scenarios. This may be attributed to the limitations of gaming controls and the need to balance tasks completion with a smooth and enjoyable gameplay experience. While commercially available VR products can provide realistic visual environments, most interactive components rely on control mechanisms. Currently, VR controllers are mostly handheld and capable of tracking the movements of only one or two single points within a three-dimensional space, imposing limitations on learning design. Although emerging technologies have to address the drawbacks of VR controller input mechanisms and enhance the transferability of acquired skills^37^, technical complexities often result in less cost-effective design solutions due to compatibility issues.

Oversimplification is a controversial issue in the design of serious games^70^. On the one hand, it leverages extrinsic motivation to drive the stimulating effects of gameplay; on the other hand, complex real-world tasks are often difficult to simulate in a virtual medium and can vary across different sociocultural contexts, posing unnecessary challenges to players and resulting in steeper learning curves. While the VR game evaluated in this study focused on breadth to assess the feasibility of immersive learning among older adults, a deeper understanding of their learning needs and the potential of immersive technologies for learning opportunities is needed. This requires the adoption of user-centered design methods and interdisciplinary efforts to create immersive learning games that align with older adults’ needs, interests, and capabilities^71^.

### Conclusions

The feasibility study presented in this paper evaluated the effectiveness of a gamified VR experience using a home-like virtual environment for the development of older adults’ independent living and cognitive skills. The qualitative findings from semi-structured interviews indicated positive responses and acceptance of the technology by participants, suggesting that the VR game was effective for its intended purposes. The study also highlighted that a familiar environment and simplified controls can enhance participant engagement with VR games, emphasizing the importance of user-friendly design tailored to the cognitive and physical abilities of older adults. Options for customizing game mechanics and control configurations can help older adults of different age groups and with varying prior experiences adapt to VR gaming, thereby increasing their acceptance and supporting a more effective learning experience.

Several limitations were identified that offer valuable insights into further research. The study’s reliance on self-reported data from participants in a specific region may limit the generalizability of the findings to other contexts. Further research incorporating quantitative methods and more diversified participant groups could help validate and extend the results of this study, providing more broadly applicable outcomes. Additionally, the trial session was limited to 20 minutes to minimize the risk of motion sickness. Future research could address this limitation by conducting longitudinal studies in care homes and community-dwelling contexts to explore whether VR gaming can aid in the development of cognitive skills that require long-term intervention, such as working memory, attention, and processing speed^72^. To develop these cognitive attributes effectively, more dedicated user experience design is needed to address accessibility and usability issues, ensuring that VR games meet the diverse learning needs and conditions of older adults^73^. Future research could also explore other areas not covered in this study, such as social integration and mental health wellbeing^74,75^.

## Data Availability

The datasets used and/or analyzed during the current study are available from the corresponding author on reasonable request.
